# Oral side effects of fluoxetine in patients with depressive disorder: A systematic review

**DOI:** 10.4317/medoral.26947

**Published:** 2025-02-15

**Authors:** M Gracia Sarrión Pérez, Yolanda Jiménez, Leticia Bagán, Jose V Bagán

**Affiliations:** 1 Lecturer. Oral Medicine, Valencia University, Valencia, Spain; 2 Senior Lecturer. Oral Medicine, Valencia University, Valencia, Spain; 3 Professor. Senior Lecturer. Oral Medicine, Valencia University, Valencia, Spain

## Abstract

**Background:**

Selective serotonin reuptake inhibitors (SSRIs) are the preferred drugs for treating depression, a condition that has become increasingly prevalent in recent years. Fluoxetine is one of the most widely used SSRIs; however, like other antidepressants, it can cause various systemic and oral adverse effects. This systematic review aimed to analyze the frequency of oral adverse effects associated with fluoxetine compared to other antidepressants.

**Material and Methods:**

A systematic review was conducted following PRISMA guidelines. A comprehensive literature search was performed in MEDLINE via PubMed, Scopus, The Cochrane Library, and Web of Science, with no date restrictions, including randomized clinical trials and observational studies. The risk of bias was assessed using the Revised Cochrane Risk-of-Bias Tool for randomized trials.

**Results:**

A total of 333 articles were identified. After removing duplicates and applying inclusion criteria, 31 randomized clinical trials were selected for analysis. Fluoxetine was primarily compared with tricyclic antidepressants, SSRIs, serotonin-norepinephrine reuptake inhibitors, and other antidepressants. The most frequently reported oral side effect was dry mouth, with prevalence rates ranging from 2.71% to 52.17%, though it was generally lower than with other antidepressants. Dysgeusia was less frequently reported, with only two studies documenting taste alterations. Oral side effects were primarily assessed through subjective patient reports, and no studies incorporated objective salivary flow measurements. Other adverse effects, such as nausea and vomiting, were commonly mentioned, but their potential oral consequences were not evaluated.

**Conclusions:**

Fluoxetine is associated with oral side effects, with dry mouth being the most frequently reported. However, data on dysgeusia remain limited, highlighting the need for further research to determine its prevalence. Given the potential impact of dry mouth on oral health and quality of life, future studies should incorporate objective salivary measurements and further investigate the clinical implications of these adverse effects.

** Key words:**Antidepressants, fluoxetine, xerostomia, dry mouth, dysgeusia.

## Introduction

Antidepressants are drugs widely used for the treatment of depression, a prevalent illness affecting approximately 280 million people worldwide. Numerous studies conducted across Europe have focused on assessing the prevalence of depression. These findings indicate that the prevalence of depression ranges between 5% and 10%, with significant variations potentially observed between countries (1). The COVID-19 pandemic has also led to an estimated 27% increase in cases (2).

The use of antidepressants has experienced a significant increase in recent decades in most countries. This increase, which began to be noticeable in the late 1980s and early 1990s, coincided with the marketing of new antidepressants, particularly selective serotonin reuptake inhibitors (SSRIs) (3). Recent studies conducted in various countries continue to confirm this growing trend. According to Alabaku *et al*. (4), high-income countries such as Spain have exhibited a significant and sustained increase in antidepressant consumption from 2014 to 2019.

The first line of treatment for depressive disorders includes selective serotonin reuptake inhibitors (SSRIs) such as fluoxetine, sertraline, paroxetine, fluvoxamine, citalopram, and escitalopram, as well as serotonin and noradrenaline reuptake inhibitors. Tricyclic antidepressants and monoamine oxidase inhibitors are used as a second line of treatment (5). Among them, fluoxetine is one of the most prescribed drugs.

SSRIs inhibit the serotonin reuptake pump, increasing postsynaptic serotonin receptor occupancy. They are selective, with relatively little affinity for other receptors, although they have secondary effects on neurotransmission involving norepinephrine and dopamine.

These drugs may cause gastrointestinal adverse effects, insomnia, headaches, and urinary retention, among others (6,7). Side effects represent the main reason for discontinuing medication, which can lead to an increased risk of relapse and chronicity of the disease.

Hyposalivation is the most frequent adverse effect at the oral level. It is a relevant factor that negatively affects the patient's oral health since saliva has essential protective functions (8). The decrease in salivary secretion directly affects the hard and soft tissues of the oral cavity, making patients more susceptible to fungal and bacterial infections, caries, and traumatic lesions (9). These pathologies can be exacerbated by neglecting oral hygiene habits, which changes in the patients' mood can cause. Furthermore, hyposialia has a negative impact on the patients' quality of life.

Although these adverse effects of antidepressants have been described in the literature in studies that evaluate the efficacy and safety of different drugs (6,7), there are practically no studies that focus on oral effects. Therefore, this study aims to analyze the frequency of oral side effects caused by fluoxetine, one of the most widely used antidepressants, in comparison to other oral antidepressants for the of treatment depression.

## Material and Methods

We followed the steps outlined in the Preferred Reporting Items for Systematic Review and Meta-Analysis (PRISMA) to conduct this systematic review and preregistered the study with PROSPERO (CRD42022350525). Ethical approval and written informed consent were not required since this was a systematic review of the published literature.

- Inclusion and exclusion criteria

This study included randomized controlled trials (RCTs) that evaluated the efficacy and safety of fluoxetine compared to other antidepressant drugs or placebo in treating depression, as well as observational studies such as case-control and cohort studies. RCT eligibility required: (1) studies conducted among adult patients diagnosed with depressive disorder using any recognized diagnostic criteria; (2) studies that evaluated any oral adverse effect associated with fluoxetine; (3) follow-up of more than one month for all the studies included. Studies involving patients taking more than one antidepressant at the same time were excluded.

- Search strategy

A systematic literature search was performed from the start of indexing without any date restrictions until May 2024 in the following electronic databases: MEDLINE via PubMed, Scopus, The Cochrane Library, and Web of Science. The search strategy used was as follows: fluoxetine AND depressive disorder AND (side effects OR adverse effects) AND (xerostomia OR dry mouth OR dysgeusia OR taste alteration). Only articles in English or Spanish were included in the searches.

- Study selection

Duplicates were removed. The searched results' titles and abstracts were initially screened by two independent authors (YJ and MGS). The reviewers' calibration was verified by assessing their agreement on evaluating the first 50 references retrieved during the searches, resulting in an excellent deal with a Kappa value of 0.871. Any disagreements were resolved by a third author (LB). Full-text articles potentially meeting the inclusion criteria were retrieved and reviewed for final inclusion in the systematic review.

- Data collection and data item

Data extraction was performed by YJ and MGS, with LB available to resolve any discrepancies.

The data of the eligible studies were summarized in tabular form, with information regarding author details, year, study design, sample type, age, sex, treatment studied (drug and dose), type of oral adverse effect, frequency of appearance, and follow-up.

- Risk of bias

Two authors (MGS and YJ) independently evaluated all included studies using the Revised Cochrane risk-of-bias tool for randomized trials (RoB2). This tool assesses six domains: 1) randomization process; 2) intervention assignment; 3) missing outcome data; 4) measurement of the outcome; 5) selection of the reported result; and 6) overall assessment.

Discrepancies in terms of risk-of-bias evaluations between raters were resolved through discussions.

## Results

- Search results

**Figure 1:**
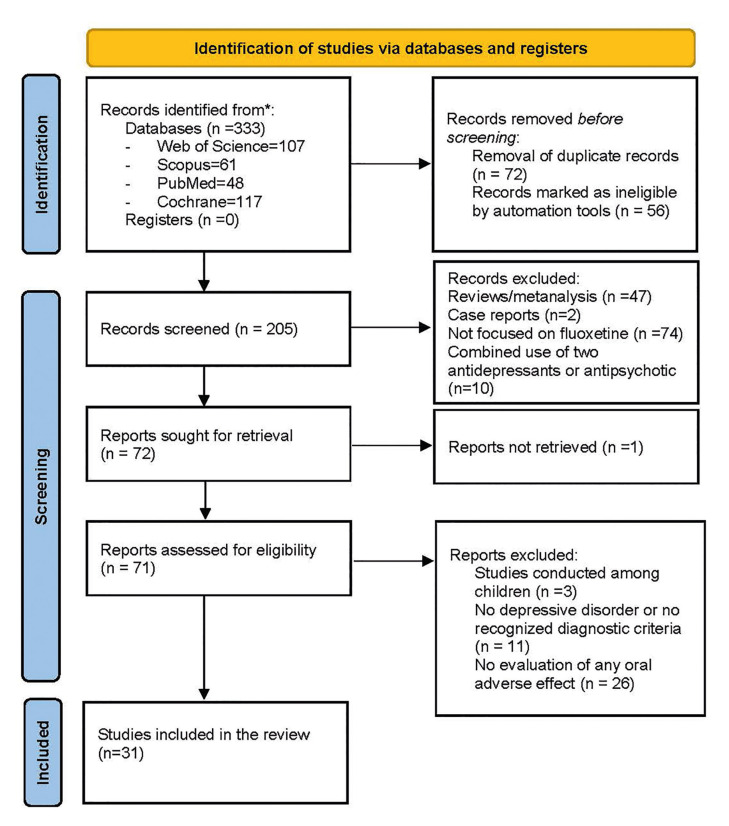
PRISMA flow diagram of study selection. PRISMA: Preferred Reporting Items for Systematic Reviews and Meta-Analyses.

The initial search yielded 333 references. After removing duplicates, 205 remained, and of these, 133 were excluded after reviewing the titles and abstracts. Finally, 31 publications met the inclusion criteria and were included in this systematic review (Fig. 1).

- Study characteristics

Thirty-one studies published between 1984 and 2021 met the inclusion criteria. All were randomized clinical trials: 26 were double-blinded, 2 were single-blinded, and 3 were open-label studies.

Sixteen studies compared the efficacy and safety of fluoxetine to different tricyclic antidepressants: four studies compared fluoxetine to amitriptyline (10-13), three to nortriptyline (14-16), five to imipramine (17-21), two to doxepin (22,23), one to tianeptine (24), and one to desipramine (25). In terms of serotonin reuptake inhibitors, citalopram (26) and vilazodone (27) were compared to fluoxetine in one study each. Reboxetine, a norepinephrine reuptake inhibitor, was compared in another (28). Several studies compared the efficacy of serotonin norepinephrine reuptake inhibitors (SNRIs) to that of fluoxetine: venlafaxine was compared in four studies (29-32) and desvenlafaxine in one (33). Mirtazapine, a noradrenergic and specific serotonergic antidepressant (NaSSA), was compared in another (34). Other drugs that were contrasted with fluoxetine include moclebomide (35), medifoxamine (37), and agometaline (38). Additionally, one study compared fluoxetine to a placebo (39) (Table 1).

- Oral side effects

**Figure 2:**
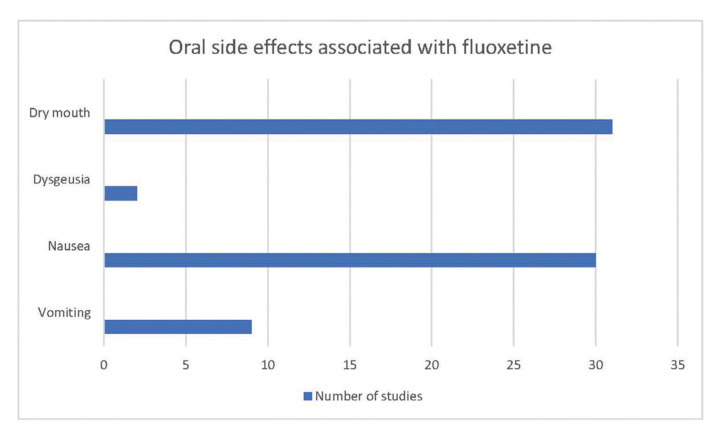
Oral side effects associated with fluoxetine according to selected studies.

The most common oral side effect associated with fluoxetine was dry mouth. Bremmer *et al*. (21) and Gosh *et al*. (33) have also reported taste perversion. Nausea and vomiting were also observed as side effects affecting the oral cavity (Fig. 2).

Most studies recorded these effects based on patients' self-report, checklists, questionnaires, or forms. Novotny *et al*. (24) and Byerley *et al*. (18) used a side effect rating scale that classified adverse effects as mild, moderate, or severe.

Xerostomia

The highest rate of xerostomia associated with fluoxetine was 52.17% in Chouinard's study (11), significantly lower than the rate associated with amitriptyline (92.85%). In contrast, the lowest rate of xerostomia (2.71%) was observed with citalopram, a serotonin reuptake inhibitor (26). In most studies, when comparing fluoxetine with other antidepressants, the rate of xerostomia was lower, except in the studies of Zhou *et al*. (31), Zisook *et al*. (39) Hashemi *et al*. (15), Lehert *et al*. (37), Lonnqvist *et al*. (36), Bougerol *et al*. (26), Díaz *et al*. (20), and Shu *et al*. (38). The frequency of dry mouth is shown in Table 1.

Fluoxetine vs. tricyclic antidepressants: When comparing amitriptyline to fluoxetine, the latter consistently had a lower rate of xerostomia in all studies. Specifically, Young *et al*. (10) reported rates of 24% for fluoxetine versus 60% for amitriptyline, Chouinard (11) reported rates of 52.17% versus 92.85%, Feighner *et al*. (12) reported rates of 13% versus 54%, and Ontiveros Sánchez de la Barquera *et al*. (13) reported rates of 15% versus 83.30%.

Hashemi *et al*. (15) has shown that fluoxetine has a higher rate of xerostomia (6.3% with fluoxetine versus 2.1% with nortriptyline). However, in contrast, Akhondzadeh *et al*. (14) found that the rate of xerostomia was three times higher with nortriptyline. Fabre *et al*. (16) also found statistically significant differences, with 20% dry mouth for fluoxetine versus 50.22% for nortriptyline.

Five studies (17-21) compared fluoxetine versus imipramine. For instance, Cohn and Wilcox (19) found that the rate of xerostomia was 25.9% with fluoxetine compared to 74.1% with imipramine. Except for one, all studies showed that fluoxetine had a lower rate of xerostomia.

Two studies comparing doxepin to fluoxetine also showed that fluoxetine was associated with a lower rate of xerostomia (22,23).

Other tricyclic antidepressants, such as tianeptine (24) and desipramine (25), were compared to fluoxetine, and in both studies, xerostomia rates were lower with fluoxetine.

Fluoxetine vs. serotonin reuptake inhibitors:When comparing serotonin reuptake inhibitors, fluoxetine was compared to citalopram and vilazodone. In the study comparing vilazodone (27) to fluoxetine, the rate of xerostomia was notably lower with fluoxetine. However, in the comparative analysis with citalopram (26), the rate was higher, although without statistically significant differences.

Fluoxetine vs norepinephrine reuptake inhibitors: In the study comparing reboxetine (28) with fluoxetine, the frequency of xerostomia was significantly lower in patients treated with fluoxetine (4.80% vs 45.5%).

Fluoxetine vs. serotonin norepinephrine reuptake inhibitors and noradrenergic and specific serotonergic antidepressants: Of the studies that compared venlafaxine to fluoxetine (30-32), all except one (31) showed that fluoxetine was associated with a lower rate of xerostomia. These differences were statistically significant. Additionally, the dry mouth sensation was lower with fluoxetine than with desvenlafaxine (33) and mirtazapine (34).

Fluoxetine vs. other antidepressants: Within the group of monoamine oxidase inhibitors, moclebamide was compared to fluoxetine. In the study by La Pierre *et al*. (35), the rate of xerostomia was significantly lower with fluoxetine. However, in the survey by Lonnqvist *et al*. (36), the sensation of dry mouth was greater with fluoxetine, although no statistical differences were found.

In a 12-month, double-blind study of 113 patients comparing medifoxamine to fluoxetine, Lehert *et al*. (37) found that xerostomia was similar to both drugs. Only 21 patients (13.54%) on fluoxetine reported dry mouth, compared to 19 (12.02%) on medifoxetamine.

Similarly, in the study by Shu *et al*. (38), dry mouth levels were very similar in both groups of patients when agometaline was compared to fluoxetine.

Fluoxetine vs. placebo: Zisook *et al*. (39) conducted a study on HIV patients to investigate whether the administration of fluoxetine, together with psychotherapy, improved depression in these patients. The study found that dry mouth occurred more frequently in the fluoxetine-treated subjects.

Fava *et al*. (40) compared the safety and efficacy of fluoxetine with St. John's Wort and a placebo. The results showed that fluoxetine was associated with a higher rate of xerostomia than the placebo (13% versus 9%), but not St John's Wort (13% versus 22%).

Dysgeusia: Only two studies described dysgeusia or taste alteration. Bremner *et al*. (21) reported a low frequency of taste alteration for both fluoxetine and imipramine (5%). However, the study by Gosh *et al*. (33) found a higher rate of 60% for fluoxetine, which was not different from desvenlafaxine (63%).

Other effects, such as nausea or vomiting that could indirectly affect the oral cavity have also been described. Thirty studies reported nausea, and nine studies reported vomiting (Fig. 2, Table 2).

- Risk of bias assessment (quality of the articles)

**Figure 3:**
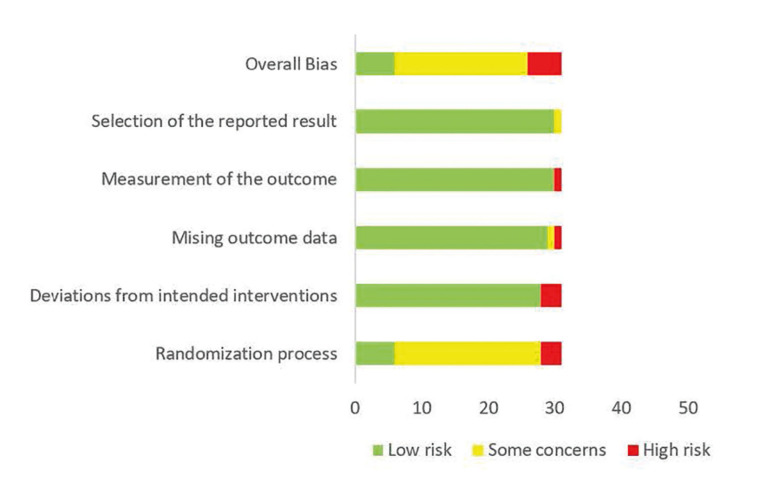
Risk-of-bias results: authors’ judgments about each risk-of-bias item across all included trials.

Fig. 3 displays the risk of bias within the five domains of RoB2 for all 31 studies, both individually and overall.

According to RoB2, 19 RCTs were rated as having some concerns regarding the risk of bias, while the RCTs by Beasley *et al*. (17), Díaz Martínez *et al*. (30), Gosh *et al*. (33), Chouinard (11), and Díaz *et al*. (20) were rated as having a high risk of bias. The remaining six RCTs were rated as having a low risk of bias.

## Discussion

The objective of this study was to analyze the frequency of oral adverse effects associated with the use of fluoxetine in patients with depression and compare it with other antidepressant drugs.

While the effect of antidepressants on flow rate reduction is well known and has been previously correlated with the plasma level of tricyclic antidepressant drugs such as imipramine and nortriptyline (41), few studies have focused on xerostomia and antidepressants. Most studies focus on the gastrointestinal side effects of fluoxetine (6), with dry mouth mentioned as another effect but not explored in depth.

Our analysis of multiple studies found a wide range of reported frequencies for dry mouth caused by fluoxetine, ranging from 2.71% to 52.17% (Table 2). Compared to other antidepressant drugs, fluoxetine generally does not have the highest incidence rate of dry mouth.

The tricyclic antidepressant class drugs were frequently compared to fluoxetine, which consistently showed lower rates of dry mouth in most studies, except for those conducted by Hashemi *et al*. (15) and Díaz *et al*. (20).

Tricyclic antidepressant drugs have a high affinity for the muscarinic receptor at the neuro-effector junctions in the gland and apparently act as competitive inhibitors of the acetylcholine released by parasympathetic nerves onto the acinar cells (42). The serotonin reuptake inhibitors do not have this affinity for muscarinic receptors. Therefore, while saliva secretion should not be affected as with tricyclic antidepressants, a substantial number of patients still report experiencing dry mouth, as noted in this review. This may be because the sensation of dry mouth is related to a reduction in flow rate and the quality of secretion, particularly the mucin content (43). According to some authors, this sensation may be caused by the interaction of these drugs with other receptors or by individual susceptibility produced by factors such as the dose used, the patient's weight, the intensity of the depression, or inherent biological variation (44).

Compared to other serotonin reuptake inhibitors and serotonin norepinephrine reuptake inhibitors, fluoxetine shows the lowest rates of xerostomia in almost all studies. Bougerol *et al*. (26) conducted a study comparing fluoxetine to citalopram. They conducted two clinical trials: one in patients with significant depression within a specialist psychiatric setting (the psychiatrist trial) and another in a general practice setting (the GP trial). The rate of xerostomia for fluoxetine was 2.71% in the GP trial, while the psychiatrist trial did not report any cases of dry mouth for either fluoxetine or citalopram.

The study conducted by Patted *et al*. (27) found that fluoxetine resulted in lower xerostomia compared to vilazodone after six weeks (3.1% versus 32.5%). However, after three weeks, the incidence of xerostomia was higher with fluoxetine (62.5% versus 32.5%), suggesting that fluoxetine produces fewer long-term adverse effects related to xerostomia compared to vilazodone, which maintains its adverse impact over time and remains undiminished.

It should be noted that the samples in some studies were very small, so the data should be interpreted with caution (25,34).

Dry mouth sensation was reported in studies using various methods, including patients' self-report (12 studies), checklists (4 studies), adverse effects questionnaires (3 studies), and forms (2 studies). Six studies did not specify how adverse effects were recorded, highlighting the challenge of comparing dry mouth frequency across studies.

On the other hand, Chouinard *et al*. (11) and Bremner (21) used the Efficacy Index-Side Effects scale, which is scored on an ascending scale of 1-4 (1 = none, 2 = does not significantly interfere with patient's functioning, 3 = significant interference, and 4 = outweighs therapeutic effect). However, side and adverse effects were recorded separately, and neither the intensity nor the effect of any of them was measured.

Only two studies reported using scales to measure side effects. Novotny *et al*. (24) used the “Arbeitsgemeinschaft für Methodik und Dokumentation Psychiatrischer Befunde scale (AMDP-5)” to assess somatic symptoms (SASS). This scale ranges from 0 to 3 (0 = absent, 1 = mild, 2 = moderate, and 3 = severe). Similarly, Byerley *et al*. (18) used a side effect rating scale in which adverse effects were rated as mild, moderate, or severe. Furthermore, all studies included in this review, regardless of using a dry mouth measurement scale or not, relied solely on patient-reported sensation to assess dry mouth, without any objective measurements, such as sialometry, to determine actual salivary flow rate and confirm a decrease in salivary secretion. Xerostomia, known as dry mouth, can occur even when salivary flow rates are normal. Therefore, it is essential to objectively determine whether there is a decrease in the salivary flow rate. Saliva can be collected under resting and stimulated conditions, and the whole saliva or parotid salivary flow rate can be measured. Moreover, not only quantitative but also qualitative changes can occur in saliva, which could be the reason for dry mouth in patients taking SSRIs.

Dry mouth may lead to several adverse effects, such as difficulty eating and speaking, dental complications, and oral diseases that can impact the patient's quality of life. None of the studies included in this analysis evaluated the potential oral manifestations of dry mouth.

Only two studies describe dysgeusia as an adverse effect of fluoxetine (21,33), but the authors did not find any differences with the compared drug. Moreover, these values are based solely on the sensation reported by the patient without any specific determinations. Other adverse effects frequently described are nausea and vomiting. However, none of these studies referred to the potential impact these adverse effects could have on hard tissues or other parts of the oral cavity in the medium or long term.

Our review has some limitations, including heterogeneity, poor accuracy in data collection on adverse effects, and limited use of measurement scales. Additionally, the short follow-up time of patients makes it difficult to recognize medium- or long-term adverse effects of these drugs. It should also be noted that the patients' check-ups were not carried out by stomatologists or dentists, which may have resulted in some oral symptoms or signs from these drugs being ignored. It is necessary to conduct studies using standardized measurement scales, examinations by oral health professionals and determination of salivary flow rate, to ensure accurate measurement of oral side effects.

## Conclusions

Dry mouth is the most common oral adverse effect associated with fluoxetine, with a frequency rate ranging from 2.71% to 52.17%. However, it is less frequent compared to other antidepressants. It is unclear whether a decrease in salivary flow rate accompanies this sensation, as the studies do not provide objective determinations.

The studies provide inadequate descriptions of dysgeusia and do not show any differences between the compared drugs. Therefore, analyzing their frequency with such limited data is impossible.

Fluoxetine frequently causes nausea and vomiting, but the studies do not describe the oral effects that these signs and symptoms may have on patients.

## Figures and Tables

**Table 1:** Frequency of dry mouth according to the studies included in the review.

	Author	Frequency of dry mouth		
		Fluoxetine	Other drugs	
Fluoxetine versus tricyclic antidepressants	Young *et al.* (10)	24%	Amitriptyline	60%
	Chouinard (11)	52.17%	Amitriptyline	92.85%
	Feighner (12)	13%	Amitriptyline	54%
	Ontiveros Sánchez de la Barquera *et al.* (13)	15%	Amitriptyline	83.30%
	Akhondzadeh *et al.* (14)	25%	Nortriptyline	75%
	Hashemi *et al.* (15)	6.3%	Nortriptyline	2.1%
	Fabre *et al.* (16)	20.40%	Nortriptyline	52.00%
	Beasley *et al.* (17)	28.6%	Imipramine	58.1%
	Byerley *et al.* (18)	19%	Imipramine	62%
			Placebo	13%
	Cohn and Wilcox (19)	25.90%	Imipramine	74.10%
			Placebo	17.20%
	Díaz *et al.* (20)	26.66%	Imipramine	23.52%
			Combined antidepressants*	25%
	Bremner (21)	5%	Imipramine	40%
	Remick *et al.* (22)	15.80%	Doxepin	67.60%
	Feighner and Cohn (23)	17%	Doxepin	57%
	Novotny *et al.* (24)	3.30%	Tianeptine	4.40%
	Schwartz and McDaniel (25)	25%	Desipramine	50%
Fluoxetine versus SSRIs	Bougerol *et al.* (26)	2.71%	Citalopram	0%
	Patted *et al.* (27)	3.10%	Vilazodone	37.50%
Fluoxetine vs NRI	Taner *et al.* (28)	4.80%	Reboxetine	45.5%
Fluoxetine versus SNRIs and NaSSA	Sheedan *et al.* (29)	24%	Venlafaxine	38%
			Placebo	9%
	Díaz Martínez *et al.* (30)	9.3%	Venlafaxine	22.9%
	Zhou *et al.* (31)	7.5%	Venlafaxine	4.5%
	Schatzberg *et al.* (32)	6%	Venlafaxine	23%
			Placebo	15%
	Gosh *et al.* (33)	40%	Desvenlafaxine	60%
	Amini *et al.* (34)	6.66%	Mirtazapine	25%
Fluoxetine vs other antidepressants	Lapierre *et al.* (35)	3%	Moclobemide	17%
	Lonnqvist *et al.* (36)	8.41%	Moclobemide	3.92%
	Lehert *et al.* (37)	13.54%	Medifoxamine	12.02%
	Shu *et al.* (38)	2.90%	Agomelatine	2.60%
Fluoxetine vs placebo	Zisook *et al.* (39)	32%	Placebo	5%
	Fava *et al.* (40)	13%	Placebo	9%

*Combined antidepressants: Amitriptyline, perphenazine, diazepam. SSRI: selective serotonin reuptake inhibitors; NRI: Norepinephrine reuptake inhibitor; SNRIs: serotonin noradrenergic reuptake inhibitors; NaSSA: noradrenergic and specific serotonergic antidepressant.

**Table 2:** Other side effects reported with fluoxetine.

Author	Oral side effect	Frequency
Young *et al.* (10)	Nausea	44%
Chouinard (11)	Nausea	56.52%
Feighner (12)	Nausea	27.27%
Akhondzadeh *et al.* (14)	Nausea	41.66%
	Vomiting	29.16%
Hashemi *et al.* (15)	Nausea, vomiting	35.4%
Fabre *et al.* (16)	Nausea	11.7%
Beasley *et al.* (17)	Nausea	14.3%
Byerley *et al.* (18)	Nausea	25%
Cohn and Wilcox (19)	Nausea	6.5%
Díaz *et al.* (20)	Nausea	13.33%
Bremner (21)	Taste change	5%
	Nausea	5%
	Vomiting	5%
Remick *et al.* (22)	Nausea	34.2%
Feighner and Cohn (23	Nausea	17%
Novotny *et al.* (24)	Nausea	6.6%
	Vomiting	2.2%
Schwartz and McDaniel (25)	Nausea	25%
Bougerol *et al.* (26)	Nausea	7.60%
	Vomiting	1.08%
Patted *et al.* (27)	Nausea	0%
Taner *et al.* (28)	Nausea	23.8%
Sheedan *et al.* (29)	Nausea	30%
Díaz Martínez *et al.* (30)	Nausea	28%
	Vomiting	4%
Zhou *et al.* (31)	Nausea	11.8%
Schatzberg *et al.* (32)	Nausea	23%
	Vomiting	2%
Gosh *et al.* (33)	Taste perversion	60%
	Nausea	60%
Amini *et al.* (34)	Nausea	46.66%
	Vomiting	6.66%
Lapierre *et al.* (35)	Nausea	22%
	Vomiting	5%
Lonnqvist *et al.* (36)	Nausea	28.97%
Lehert *et al.* (37)	Nausea	30.96%
Shu *et al.* (38)	Nausea	11%
Zisook *et al.* (39)	Nausea	48%
Fava *et al.* (40)	Nausea	9%
